# Anoxygenic photoautotrophy driven by humus and microplastics in a photosynthetic bacterium

**DOI:** 10.1093/ismeco/ycaf067

**Published:** 2025-04-18

**Authors:** Yutong Li, Kongyuang Qu, Jianming Yang, Shuguang Wang, Zhen Yan

**Affiliations:** Shandong Key Laboratory of Water Pollution Control and Resource Reuse, School of Environmental Science and Engineering, Shandong University, No. 72 Binhai Road, Jimo District, Qingdao, Shandong 266237, China; Shandong Key Laboratory of Environmental Processes and Health, School of Environmental Science and Engineering, Shandong University, No. 72 Binhai Road, Jimo District, Qingdao, Shandong 266237, China; Shandong Key Laboratory of Water Pollution Control and Resource Reuse, School of Environmental Science and Engineering, Shandong University, No. 72 Binhai Road, Jimo District, Qingdao, Shandong 266237, China; Shandong Key Laboratory of Environmental Processes and Health, School of Environmental Science and Engineering, Shandong University, No. 72 Binhai Road, Jimo District, Qingdao, Shandong 266237, China; Energy-Rich Compounds Production by Photosynthetic Carbon Fixation Research Center, Shandong Key Lab of Applied Mycology, College of Life Sciences, Qingdao Agricultural University, No. 700 Changcheng Road, Chengyang District, Qingdao, Shandong 266109, China; Shandong Key Laboratory of Water Pollution Control and Resource Reuse, School of Environmental Science and Engineering, Shandong University, No. 72 Binhai Road, Jimo District, Qingdao, Shandong 266237, China; Shandong Key Laboratory of Environmental Processes and Health, School of Environmental Science and Engineering, Shandong University, No. 72 Binhai Road, Jimo District, Qingdao, Shandong 266237, China; Sino-French Research Institute for Ecology and Environment (ISFREE), School of Environmental Science and Engineering, Shandong University, No. 72 Binhai Road, Jimo District, Qingdao, Shandong 266237, China; WeiHai Research Institute of Industrial Technology of Shandong University, No. 180 Wenhua Xilu, Weihai 264209, China; Shandong Key Laboratory of Water Pollution Control and Resource Reuse, School of Environmental Science and Engineering, Shandong University, No. 72 Binhai Road, Jimo District, Qingdao, Shandong 266237, China; Shandong Key Laboratory of Environmental Processes and Health, School of Environmental Science and Engineering, Shandong University, No. 72 Binhai Road, Jimo District, Qingdao, Shandong 266237, China

**Keywords:** CO_2_ fixation, extracellular electron transport, photoexcitation, *Rhodopseudomonas palustris*, sunlit anoxic environments

## Abstract

Humus and microplastics are recalcitrant organics in soils and aquatic systems, and their role in the geochemical cycling of elements remains elusive. Herein, we have identified a new mechanism by which humus and microplastics participate in anoxic carbon cycling. We demonstrated that the photoexcitation of 5–30 mg/l of humic acid or fulvic acid, two major fractions of humus, can drive CO_2_ fixation and enable the photoautotrophic growth of a photosynthetic bacterium, *Rhodopseudomonas palustris*. This process was enhanced by 10.69%–144.87% upon the addition of 100 mg/l of poly(lactic acid) or poly(ethylene terephthalate). Mechanistic investigations demonstrated that the microplastics act as sacrificial quenchers during humus photoexcitation, leading to their depolymerization. Transcriptomic analyses revealed high expression of genes encoding extracellular electron uptake pathways including extracellular cytochrome *c* and its oxidases in the photoautotrophic growth of *R. palustris*. This study expands our understanding of how humus and microplastics are involved in the biogeochemical cycling of carbon and sheds light on how they impact the CO_2_ dynamic fluxes in sunlit anoxic environments.

## Graphical Abstract

**Graphical Abstract:**
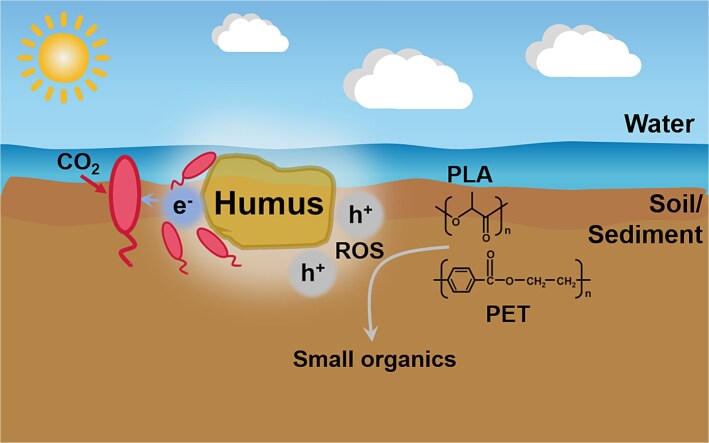

## Introduction

Humus, a term used to indicate humic substances, is the highly transformed component of nonliving natural organic matter, representing a major carbon reservoir in various terrestrial and aquatic environments [1]. It is estimated that humus makes up ~70% of the organic matter in terrestrial soil and 25%–90% of the dissolved organic matter in diverse freshwater ecosystems [2–4]. While humus is generally resistant to microbial degradation, it plays a crucial role in biogeochemical cycling by acting as an electron acceptor or donor for anaerobic microbial respiration owing to its favorable redox properties [4, 5]. The ability of humus to act as an electron acceptor for anaerobic respiration in soil electrobacteria was initially demonstrated [6]. Subsequently, the fact that electrobacteria use reduced humus as an electron donor for the reduction of electron acceptors with a more positive redox potential was demonstrated [7]. For instance, several species from *Geobacter* and *Shewanella* were found to be capable of oxidizing reduced humus or its analogue with nitrate or fumarate as an electron acceptor [7]. Besides, humus has been recently found to serve as an electron acceptor for the respiratory growth of methanogenic archaea, enhancing fermentative methanogenic growth [8].

In addition to its redox properties in dark environments, humus exhibits photoelectric properties due to the presence of photoactive functional groups (e.g. aromatic groups), which release electrons upon photoexcitation [9, 10]. The photoexcitation of humus has been shown to play a role in the biogeochemical processes of elements in sunlit anoxic environments, such as the water–sediment interface of saturated soil [11]. Studies have demonstrated that photoexcited humus can act as an electron donor to power nitrate reduction by denitrifying bacteria, thus influencing the biogeochemical cycling of nitrogen [9]. However, there may be other biogeochemical processes attributed to the photoexcitation of humus that have not been fully recognized. Anoxygenic photosynthesis, carried out by ancient photosynthetic bacteria, serves as a significant means for carbon dioxide (CO_2_) fixation in anoxic environments [12–14]. These bacteria proliferate in sunlit anoxic environments, utilizing various electron donors, including dihydrogen, hydrogen sulfide, thiosulfate, and elemental sulfur for the reductive assimilation of CO_2_, yet the potential of photoexcited humus as an electron donor for driving anoxygenic photosynthesis has not been thoroughly explored [15].

Microplastics, defined as plastic particles with a diameter of <5 mm, are derived from both the leakage of pre-existing small plastics and the fragmentation of large plastics items [16]. As a newly recognized category of environmental pollutants, microplastics are now pervasive in both terrestrial and aquatic ecosystems, where they may play a role in the biogeochemical cycling of various elements [16]. The mechanisms by which microplastics influence the biogeochemical carbon cycling are intricate, but two primary pathways have been identified: (i) microplastics can modify the structure and physicochemical properties of soil and sediment, thereby indirectly affecting microbial activities and carbon cycling [17–19]; (ii) certain microplastics and the dissolved organic matter they release can be metabolized by microorganisms, directly or indirectly influencing carbon cycling and CO_2_ emissions [20, 21]. To address the escalating issue of microplastic pollution, a variety of microplastic depolymerization technologies have been developed, among which photocatalytic depolymerization has proven to be particularly effective [22, 23]. In artificial photocatalysis, the generation of photoexcited holes (h^+^) and reactive oxygen species (ROS) is inevitable [24]. These reactive species can oxidize microplastics, especially those containing heteroatoms other than carbon and hydrogen [25]. During this process, microplastics are depolymerized into small molecules, which act as sacrificial reagents to quench the generated h^+^ and ROS [26]. Similarly, the photoexcitation of humus is a natural photocatalytic process and also produces h^+^ and ROS. Given that microplastics can serve as sacrificial quenchers for artificial photocatalysts, this raises the question of whether they also function as quenchers in natural photocatalytic processes, such as the photoexcitation of humus. This potential role could imply that microplastics are involved in CO_2_ fixation through anoxygenic photosynthesis, a hypothesis that warrants further investigation.

**Figure 1:**
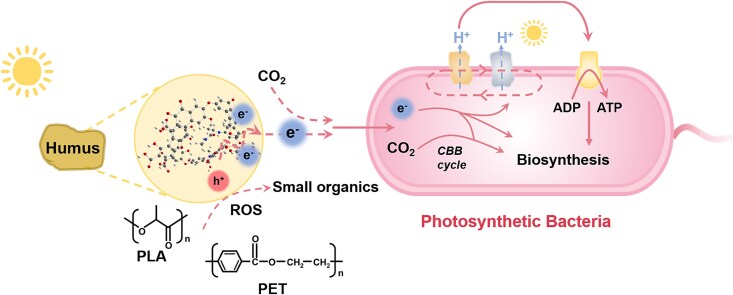
Illustration for the anoxygenic photoautotrophy of *R. palustris* driven by humus and microplastics. The photoexcitation of humus can drive CO_2_ fixation and enable the photoautotrophic growth of *R. palustris*, which is enhanced by the presence of PLA or PET. Role of microplastics as a sacrificial quencher for the photoexcitation of humus is proposed.

This study focused on investigating the role of humus and microplastics in anoxygenic photosynthesis using the photosynthetic bacterium *Rhodopseudomonas palustris*. This bacterium, commonly found in soil and aquatic environments, is capable of photoautotrophic growth using CO_2_ as its sole carbon source [27]. Since plastics containing heteroatoms are more susceptible to ROS attack, poly(lactic acid) (PLA) and poly(ethylene terephthalate) (PET) were selected for this study. The findings demonstrate that photoexcited humus can drive CO_2_ fixation, enabling the photoautotrophic growth of *R. palustris*. In addition, both types of microplastics were found to enhance this process by serving as sacrificial quenchers for the photoexcitation of humus (Fig. 1). This study also elucidates the redox interaction mechanisms between humus, *R. palustris*, and microplastics and discusses the ecological implications of these findings.

## Materials and methods

### Characterization of humic acid/fulvic acid

Fulvic acid (FA, CAS: 479-66-3) and humic acid (HA, CAS: 1415-93-6) stock solutions (30 g/l) were prepared, and the pH was adjusted to 7.0 with 3 M HCl. FA and HA were filter-sterilized (0.22 μm) and stored in the dark at 4°C. Ultraviolet–visible spectrophotometry (UV-2100, Shimadzu, Japan) was used to record the adsorption spectra of FA and HA (30 mg/l). Electron paramagnetic resonance (EPR, Bruker EMX PLUS, German) was used to determine the electron content in 100 μl of HA/FA (30 mg/l) with 2 μl of 2,2,6,6-tetramethylpiperidine-1-oxyl (TEMPO) as the spin probe at 0 and 10 min of light exposure under ambient air conditions. Photocurrent (*I–t*) curves of FA and HA were obtained by constructing a three-electrode system in which a glassy carbon electrode was used as the working electrode, an Ag/AgCl electrode was used as the reference electrode, and a platinum wire was used as the counter electrode. Phosphate-buffered saline (0.1 M) with 0.1 M KCl aqueous solution (pH 7.0) was used as the electrolyte. The FA and HA stock solutions were added to final concentrations of 10, 20, or 30 mg/l. The light source was a xenon lamp equipped with an Air Mass (AM) 1.5 filter.

### Bacterial strain and culture conditions

*Rhodopseudomonas palustris* CGA009 was cultured under photoautotrophic conditions at 30°C with N_2_/CO_2_ (v/v = 80:20) under illumination in defined mineral photosynthetic medium (PM) as described elsewhere [28]. The FA and HA stock solutions were added to the PM at final concentrations of 10, 20, or 30 mg/l. PLA and PET microplastics were added to the PM at final concentrations of 100, 500, or 1000 mg/l. All growth experiments were carried out with the simulated daylight (~7000 lux) unless otherwise noted.

*Rhodopseudomonas palustris* culture density was quantified spectrophotometrically at 680 nm (OD_680_). Consumption of CO_2_ was determined by a gas chromatograph (GC-7820, Lunan Technique, China). N_2_ was used as the carrier gas. For each analysis, gas samples (1 ml) were taken from the headspace of the cultures with a micro-injector and manually injected into the gas chromatograph. CO_2_ released into the headspace was measured as the dissolved CO_2_ content of the medium after adding an equal volume of 3 M HCl to the medium. The CO_2_ fixation rate was calculated as follows:


(1) \begin{equation*} {\mathrm{CO}}_2\ \mathrm{fixation}\%=\frac{{\mathrm{n}}_{{\left({\mathrm{CO}}_2\right)}_{0\mathrm{d}}}-{\mathrm{n}}_{{\left({\mathrm{CO}}_2\right)}_{2\mathrm{d}}}-{\mathrm{n}}_{{\left({\mathrm{CO}}_2\right)}_{\mathrm{dissolved}}}}{{\mathrm{n}}_{{\left({\mathrm{CO}}_2\right)}_{0\mathrm{d}}}} \end{equation*}

where *n*_(CO2)0d_ and *n*_(CO2)2d_ (mol) represent the CO_2_ content of the headspace on 0 and 2 d, and *n*_(CO2)dissolved_ (mol) is the dissolved CO_2_ in the medium.

### Photoelectric analysis of *R. palustris* + humic acid and *R. palustris* + fulvic acid

The charge separation and transfer resistance of *R. palustris* + FA and *R. palustris* + HA were measured by *I–t* curves and electrochemical impedance spectroscopy (EIS) using the electrochemical workstation (CHI600E, China). The cell suspensions (200 μl, OD_680_ = 0.1) were added to the indium tin oxide conductive glass slide (1 × 1 cm^2^) in 10 drops and then dried to a film in air for 2 h to construct a working electrode. Platinum sheet and an Ag/AgCl electrode were employed as counter and reference electrodes, respectively. Phosphate-buffered saline (0.1 M, prepared from NaH_2_PO_4_ and Na_2_HPO_4_) with 0.1 M KCl (pH 7.0) was used as the electrolyte. The *I–t* was measured under a light on/off cycle of 30/30 s at a potential of +600 mV vs. Ag/AgCl. The frequency range of the EIS measurement was 10 000–0.01 Hz. The EIS data were fitted using ZSimpWin software to an equivalent circuit comprising solution resistance (*R_s_*), resistance for charge transfer across the interface (*R_ct_*), and space charge capacitance (*Q*). The light source was a xenon lamp equipped with an AM 1.5 filter.

## Microplastic depolymerization

EPR was used to detect the h^+^ signal by adding 2 μl of TEMPO as a spin probe to 100 μl of *R. palustris* + FA or *R. palustris* + HA (OD_680_ = 0.1) with and without PLA and PET at 0 and 10 min of light exposure under ambient air conditions.

Scanning electron microscopy (SEM, Quanta 250 FEG, FEI, USA) was used to observe the morphology of PLA and PET with and without *R. palustris* + FA or *R. palustris* + HA. The microplastics were collected by centrifugation (5000 *g*, 15 min), washed three times with 0.5% NaCl, and fixed in 2.50 vol% glutaraldehyde overnight. The samples were washed in a series of aqueous ethanol (30%, 50%, 70%, 80%, 90%, 100%, and 100%), 15 min per wash. Finally, the samples were dried in a critical point dryer and gold sputter–coated before SEM observation.

To completely remove microbes and other substances, PLA/PET was washed with 2% sodium dodecyl sulfate in deionized water and dried at ambient temperature. The molecular weight distributions of PLA and PET were measured by gel permeation chromatography (GPC, PL-GPC50, Agilent, USA). The mobile phases used for PLA and PET were trichloromethane and hexafluoroisopropanol, respectively. The flow rate was 1 ml/min. Samples were filtered using a syringe filter with a polytetrafluoroethylene membrane (pore diameter 0.45 μm) and then injected for each analysis. Polystyrene standards with narrow molar mass dispersities were used for calibration curve.

The surface functional groups of PLA and PET were analyzed by Fourier transform infrared microscopy (FTIR microscope, Nicolet iN10, ThermoFisher Scientific, USA) at 4000–400 cm^−1^. The absorbance ratio of the carbonyl groups relative to the methylene peaks was referred to as the carbonyl index (CI) value, which was the ratio of the absorbance of 1751–1454 cm^−1^ for PLA and 1720–1407 cm^−1^ for PET [29].

The elemental composition was investigated by X-ray photoelectron spectroscopy (XPS, K-Alpha, Thermo Fisher Scientific, USA) with monochromatic Al Kα radiation (1486.6 eV) using a K-Alpha X-ray photoelectron spectrometer. The peak deconvolution for C 1 s was made using the Avantage software.

The ^1^H Nuclear Magnetic Resonance Spectroscopy (NMR) spectra were acquired using a Bruker Avance NEO 600 spectrometer. All samples, including those subjected to pyrolysis (180°C, 1 h) and FA/HA treatments, were prepared in D_2_O with a sample concentration of 1.67 mg/ml for analysis.

### Transcriptomics

*Rhodopseudomonas palustris* grown without and with FA or FA + PLA was harvested at 3 d, rapidly frozen in liquid nitrogen, and stored at −80°C. Total RNA was extracted using TRIzol-based methods (Invitrogen). The RNA quality was assessed using a bioanalyzer (Agilent2100). Libraries were constructed using the Truseq Stranded RNA sample prep kit (Illumina). The libraries were sequenced using the Truseq SBS Kit (300 cycles) (Illumina). Genes meeting *P* < .05 and |log_2_FC| > 1 criteria were identified as DEGs.

### Calculation of quantum yield under CO_2_ fixation and molar production of biomass

The quantum yield (QY) was calculated by comparing the number of electrons accepted by CO_2_ fixation with the measured number of incident photons. The biomass formula of *R. palustris* was CH_1.8_N_0.18_O_0.38_, where the valence state of C was −0.5, so producing one CH_1.8_N_0.18_O_0.38_ molecule requires 4.5 electrons. The QY% was calculated as follows [30]:


(2) \begin{align*} \mathrm{QY}\%&=\frac{\mathrm{the}\ \mathrm{number}\ \mathrm{of}\ \mathrm{electron}\ \mathrm{accepted}\ \mathrm{by}\ {\mathrm{CO}}_2}{\mathrm{the}\ \mathrm{number}\ \mathrm{of}\ \mathrm{incident}\ \mathrm{photons}}\times 100\%\nonumber \\ &=\frac{10^9\left(v\times{\mathrm{N}}_{\mathrm{A}}\times \mathrm{K}\right)\times \left(h\times \mathrm{c}\right)}{\left(\mathrm{I}\times \mathrm{A}\times \mathrm{\lambda} \right)}\times 100\% \end{align*}

where *v* is the reaction ration (mol/s), *N*_A_ is the Avogadro number (6.02 × 10^23^ mol^−1^), *K* is the electron transfer number, *h* is the Plank constant (6.62 × 10^−34^ J·s), *c* is the speed of light (3.0 × 10^8^ m·s^−1^), *I* is the optical power density (W·m^−2^), *A* is the incident light area (60.45 cm^2^), and λ is the incident light wavelength (405 nm).

The molecular weight of biomass was 22.426 g/mol according to the biomass formula. The molar production of biomass was calculated by [31, 32]:


(3) \begin{align*} & \mathrm{Molar}\ \mathrm{production}\ \mathrm{of}\ \mathrm{biomass} \nonumber \\ &\quad =\frac{\left({\mathrm{OD}}_{2\mathrm{d}}-{\mathrm{OD}}_{0\mathrm{d}}\right)\times \left(\frac{\mathrm{cell}\ \mathrm{number}}{\mathrm{mL}}\right)\times \mathrm{cell}\ \mathrm{weight}}{\mathrm{Molecular}\ \mathrm{weight}\ \mathrm{of}\ \mathrm{biomass}} \end{align*}

where OD_0d_ and OD_2d_ represent the optical density of on 0 and 2 d, cell number/ml is 8 $\times$ 10^8^ cell/ml/OD, cell weight is 10^−12^ g/cell, and the molecular weight of biomass is 22.426 g/mol.

### Statistical analysis

In all experiments, at least three independent replications were performed (*n* ≥ 3). All statistical significances for differences were analyzed by one-way Analysis of Variance (ANOVA) using Statistical Product and Service Solutions (SPSS) 26.0 (IBM Corp., Armonk, NY, USA). The significance levels were expressed at ^*^P < .05 and ^*^^*^P < .01. The comparative factors and “*P*-value” for statistical tests are labeled in the legend of each figure.

## Results

### Roles of humus and microplastics in the photoautotrophic growth of *R. palustris*

In this study, two major fractions of humus, FA and HA, were utilized. Both FA and HA exhibited photochemical properties, as characterized by ultraviolet-visible and EPR spectra. It was observed that both FA and HA absorbed light at 250–600 nm (Fig. S1A), and h^+^ was generated for both FA and HA upon 10 min of illumination (Fig. S1B and C). Current measurements indicated that 30 mg/l of FA and HA dissolved in an electrolyte solution were capable of producing photocurrents of 1.5 and 0.6 μA/cm^2^, respectively (Fig. S1D and E).

**Figure 2:**
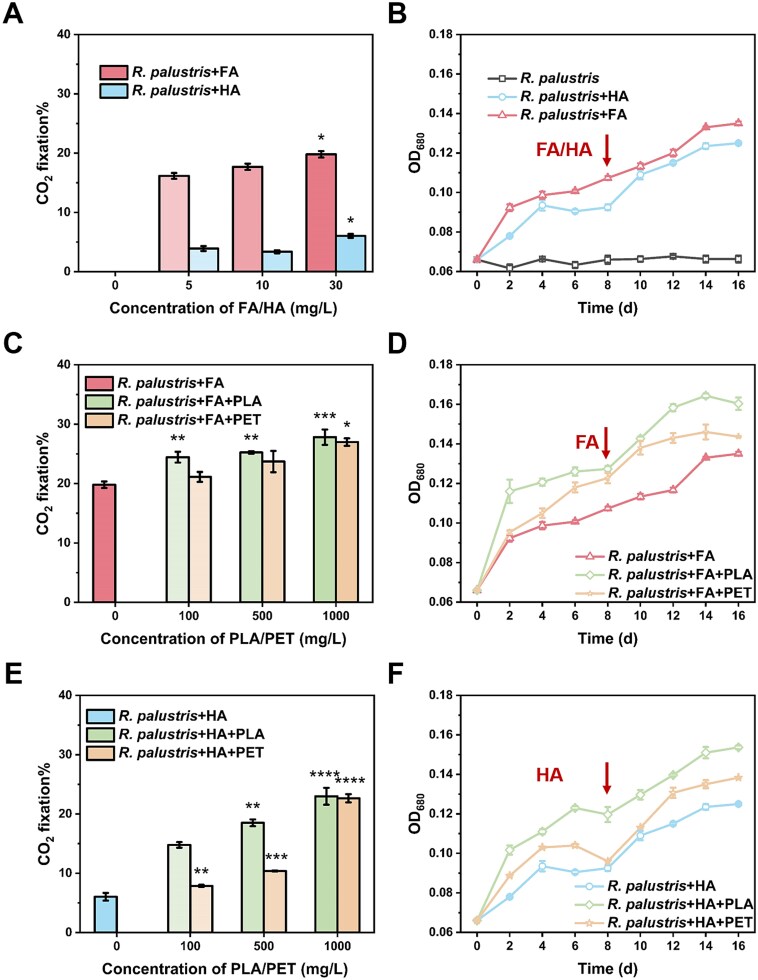
Roles of humus and microplastics on the photoautotrophic growth of *R. palustris*. (A) Effect of FA or HA on the CO_2_ fixation during the photoautotrophic growth of *R. palustris* at Day 2. (B) Effect of FA or HA on the photoautotrophic growth of *R. palustris*. (C) Effect of FA with PLA or PET on the CO_2_ fixation during the photoautotrophic growth of *R. palustris* at Day 2. (D) Effect of FA with PLA or PET on the photoautotrophic growth of *R. palustris*. (E) Effect of HA with PLA or PET on the CO_2_ fixation during the photoautotrophic growth of *R. palustris* at Day 2. (f) Effect of HA with PLA or PET on the photoautotrophic growth of *R. palustris*. Arrows represent the re-addition of fresh FA or HA. Data are represented as mean ± SD (*n* = 3). ^*^P < .05, ^*^^*^P < .01, ^*^^*^^*^P < .005, ^*^^*^^*^^*^P < .001.

Furthermore, the capability of FA and HA as electron donors for the photoautotrophic growth of *R. palustris* was evaluated. *Rhodopseudomonas palustris* was cultured in a photoautotrophic manner with 20% CO_2_ in the headspace as the sole carbon source. Any previously known electron donors that could support the growth of *R. palustris* were omitted from the medium. Supplementation of the medium with 5–30 mg/l of FA or HA drove *R. palustris*-mediated CO_2_ fixation (Fig. 2A). Notably, FA-driven CO_2_ fixation efficiency (19.08 ± 0.55%) was significantly higher than HA-driven CO_2_ fixation efficiency (6.04 ± 0.33%), consistent with the results of current intensities generated by the photoexcitation of FA and HA (Fig. S1F). In addition, the photoautotrophic growth of *R. palustris* was observed upon supplementation of the medium with 30 mg/l of FA or HA, as evidenced by the measurement of optical density at 680 nm. The sustainable growth of *R. palustris* was maintained by reading fresh 30 mg/l FA or HA when growth ceased at Day 8 (Fig. 2B). It should be noted that the growth rate observed within the initial 2-d period was significantly higher compared to subsequent stages. This phenomenon can be primarily attributed to the photobleaching effect of either HA or FA, which led to a substantial reduction in the photoexcitation currents under prolonged light exposure [33]. Although subject to uncertainties and undetermined error upon the addition of HA or FA, the results suggest that humus under illumination can serve as an electron donor for the respiratory chain of *R. palustris*, thereby enabling its photoautotrophic growth. The possibility of potential degradation products of FA or HA being used as a carbon source for supporting the growth of *R. palustris* in the absence of CO_2_ was ruled out (Fig. S2A), indicating the significance of humus in supporting the photoautotrophic growth of *R. palustris*.

To further explore the impact of microplastics on the photoautotrophic growth of *R. palustris*, we introduced two types of microplastics—PLA and PET—at a size of ~250 μm into the medium with FA or HA as an electron donor, respectively. The addition of 100–1000 mg/l of PLA or PET significantly increased *R. palustris*-mediated CO_2_ fixation by 10.69%–280.56% (Fig. 2C and E). Moreover, supplementing with 100 mg/l of PLA or PET significantly enhanced the photoautotrophic growth of *R. palustris*, and reintroducing FA or HA on Day 8 promoted sustained growth (Fig. 2D and F). Notably, *R. palustris* was unable to grow without FA or HA as an electron donor, indicating that the microplastics were not directly used as a carbon source through degradation by *R. palustris* (Fig. S2B). However, supplementation with PLA or PET pretreated at 180°C for 1 h sustained the growth of *R. palustris* in the absence of FA or HA (Fig. S2B). The pyrolytic products of PLA and PET contain lactate and ethylene glycol, respectively, which were detected by NMR (Fig. S2C and D), and the two pyrolytic products have been reported to be carbon sources for heterotrophic growth of *R. palustris* [34, 35].

### Mechanism of humus and microplastic-driven photoautotrophic growth of *R. palustris*

**Figure 3:**
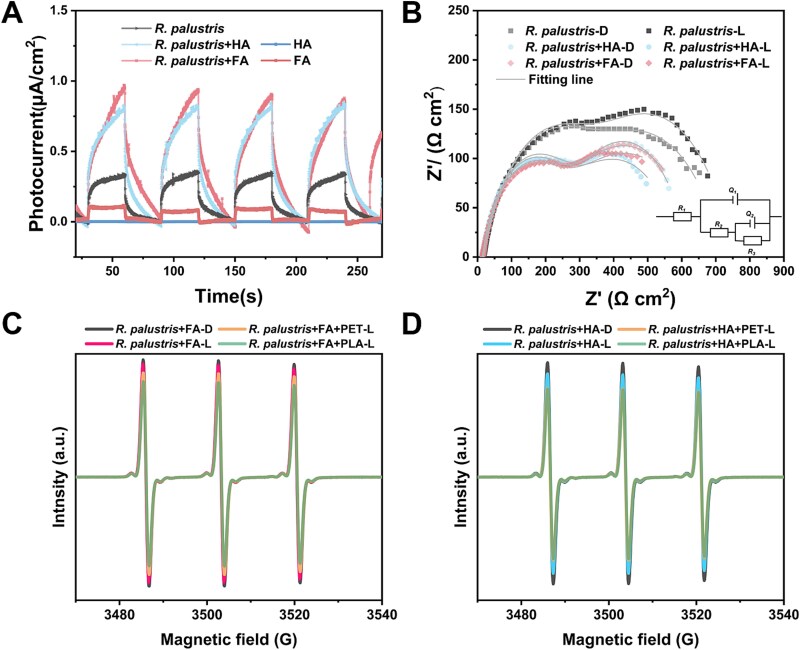
Photoelectric characterization of the *R. palustris* + FA or + HA with PLA or PET. (A) *I-t* curves with a light on/off cycle (30/30 s); (B) Nyquist plots of *R. palustris* + FA or + HA under light or dark conditions. (working electrode: ITO conductive glass slide (1 × 1 cm^2^) with *R. palustris* suspensions; counter electrode: platinum sheet; reference electrode: Ag/AgCl electrode; light source: xenon lamp equipped with AM 1.5 filter). EPR spectra for h^+^ of (C) *R. palustris* + FA and (D) *R. palustris* + HA with the microplastics or not after 10 min of light exposure under air conditions (light source: xenon lamp).

To demonstrate the occurrence of photoexcited electron transfer between humus and *R. palustris*, cultures of *R. palustris* amended with FA (*R. palustris* + FA) or HA (*R. palustris* + HA) were sampled and loaded onto an indium tin oxide conductive glass slide, which was used as the working electrode for the measurements of photocurrent. Amperometric *I–t* curves showed that HA or FA alone exhibited nearly no photocurrent intensity, while the photocurrent intensity of *R. palustris* + FA or *R. palustris* + HA immediately increased to 1.0 μA/cm^2^ under illumination, a value much higher than that of *R. palustris* (Fig. 3A), indicating electrons produced from the photoexcited *R. palustris* + FA or + HA. Electrochemical impedance spectroscopy was performed to evaluate the photoexcited electron transfer efficiency at the interface of *R. palustris* + FA or *R. palustris* + HA. If there is photoexcited electron transfer in *R. palustris* + FA or *R. palustris* + HA, the interfacial resistance (*Rct*) would decrease. As expected, the *Rct* of *R. palustris* + FA and *R. palustris* + HA decreased from 268.8 and 299.8 Ω·cm^2^ in the dark to 254.7 and 271.5 Ω·cm^2^ under the illumination, respectively, while the *Rct* of *R. palustris* alone increased from 382.6 Ω·cm^2^ in the dark to 389.9 Ω·cm^2^ under the illumination, indicating the enhanced transfer of photoelectrons and higher electronic conductivity of *R. palustris* + FA or *R. palustris* + HA under light irradiation. (Fig. 3B and Table S1). Furthermore, to assess the role of microplastics in the photoexcited *R. palustris* + FA or *R. palustris* + HA, the EPR spectra of TEMPO reacted with *R. palustris* + FA or *R. palustris* + HA were monitored. TEMPO is a spin-label molecule with a stable triplet EPR spectrum, and the EPR signal will diminish after TEMPO reacts with photoexcited h^+^. The reduction of the EPR signal was observed for *R. palustris* + FA or *R. palustris* + HA amended with PLA or PET compared to that of the corresponding group without PLA or PET, indicating that the microplastics played a quenching role (Fig. 3C and D).

**Figure 4:**
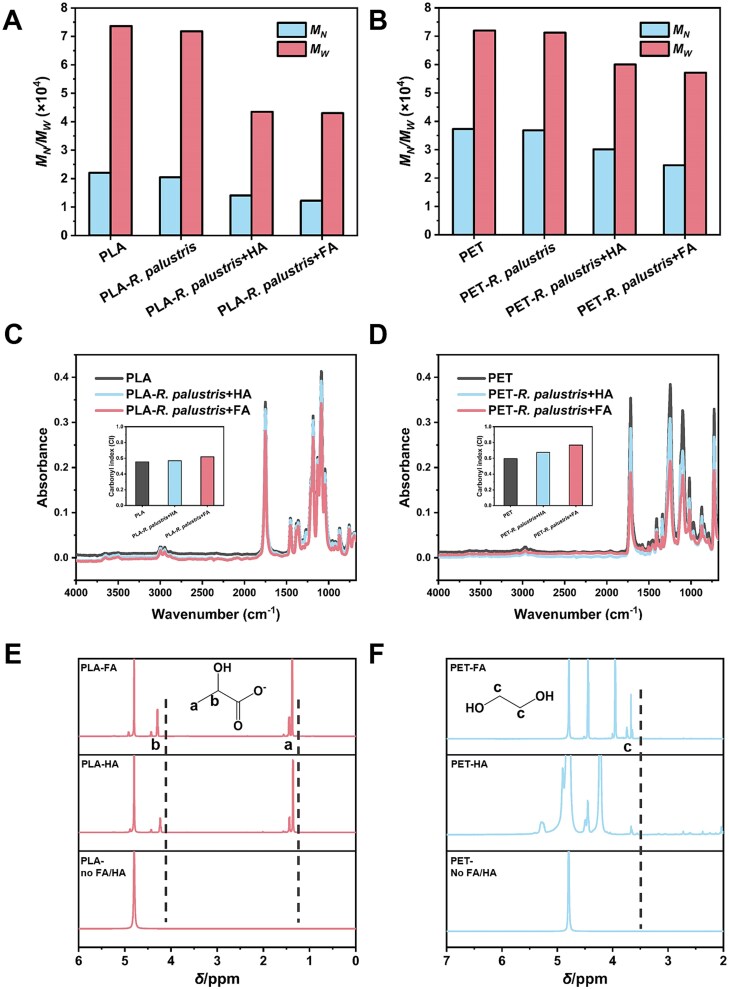
Analysis for the depolymerization of PLA and PET during the photoexcitation of humus. The molecular weight of (A) PLA and (B) PET incubated with FA or HA under illumination (*M*_N_ is the number-average molecular weight; *M*_W_ is the weight-average molecular weight); FTIR spectra of (C) PLA and (D) PET incubated with FA or HA under illumination (the inset images show the CI of PLA and PET with various treatment); ^1^H NMR spectra of (E) PLA and (F) PET after photoreforming with FA or HA.

When *R. palustris* + FA or *R. palustris* + HA were used as sacrificial quenchers under illumination, the photogenerated ROS oxidized the microplastics, leading to their depolymerization. To assess this, the molecular weights of PLA and PET before and after photoexcitation were characterized by GPC. The number-average molecular weight (*M*_N_)/the weight-average molecular weight (*M*_W_) of PLA decreased from 73 645/22065 to 43 004/12235 and 43 455/14074, respectively, when incubated with *R. palustris* + FA and *R. palustris* + HA under illumination (Fig. 4A and Table S2). Similarly, the *M*_W_*/M*_N_ of PET decreased from 72 016/37308 to 57 157/24502 and 60 023/30137 for incubation with *R. palustris* + FA and *R. palustris* + HA, respectively (Fig. 4B and Table S2). FTIR spectra were used to analyze the structural changes in the microplastics before and after incubation with *R. palustris* + FA and *R. palustris* + HA under illumination. The CI, which indicates carbonyl abundances during microplastic depolymerization, was calculated from the absorbance ratio of the carbonyl groups to methylene peaks in the FTIR spectra. The CI of PLA increased from 0.55 to 0.57 and 0.62 after incubation with *R. palustris* + FA and *R. palustris* + HA under illumination, respectively, while the CI of PET increased from 0.59 to 0.67 and 0.76 after incubation with *R. palustris* + FA and *R. palustris* + HA under illumination, respectively (Fig. 4C and D). In addition, compared with the untreated PLA and PET, the atomic percentage of –C=O– and O/C decreased with *R. palustris* + FA and *R. palustris* + HA treatment (Fig. S3 and Table S2). Finally, the depolymerization of PLA and PET was confirmed through ^1^H NMR analysis, which detected their respective depolymerization products, lactate and ethylene glycerol, in the FA or HA treatment groups (Fig. 4E and F). Analysis of the microplastics morphology showed that there were cavities and cracks on the surface of the depolymerized microplastics (Fig. S4).

### Transcriptomics analyses of humus and microplastic-driven photoautotrophic growth of *R. palustris*

Sodium thiosulfate is the most commonly used electron donor for laboratory photoautotrophic batch–cultured *R. palustris*. When added at 4 mM as a sole electron donor, sodium thiosulfate drove CO_2_ fixation and enabled the photoautotrophic growth of *R. palustris* (Fig. S5). Transcriptomic analyses were performed to identify differences in gene expression during photoautotrophic growth using thiosulfate and FA as electron donors, as well as differences in FA-driven photoautotrophic growth with and without PLA as a sacrificial quencher. Differentially expressed genes (DEGs) were identified through univariate statistical analysis. The results showed that a total of 893 and 239 genes were upregulated and downregulated, respectively, in the FA-amended culture compared to the thiosulfate-amended culture, indicating metabolic profile variation in photoautotrophic growth with different electron donors (Fig. S6A). In addition, nine genes were upregulated and eight genes were downregulated in the FA- and PLA-amended culture compared to the FA-amended culture, suggesting that PLA as an extracellular quencher did not significantly impact the metabolic profile of the photoautotrophic growth (Fig. S6B). The DEGs between the FA and thiosulfate groups were enriched in various metabolic pathways, such as energy and biosynthetic metabolisms, which are directly involved in photophosphorylation and CO_2_ fixation (Fig. S6C). Notably, genes encoding light-harvesting proteins and enzymes involved in the Calvin–Benson–Bassham cycle were significantly upregulated in the thiosulfate group compared to the FA group (Table S3), consistent with the superior performance of photoautotrophic growth using thiosulfate as an electron donor (Fig. S5). Genes encoding sulfite oxidase (Sox), responsible for thiosulfate oxidation, were also significantly upregulated in the thiosulfate group compared to the FA group (Table S3). Furthermore, genes encoding extracellular electron transfer proteins, including extracellular cytochrome *c*, membrane-bound cytochrome *c* oxidases, and flagellar proteins, were significantly upregulated in the FA group compared to the thiosulfate group, suggesting their role in accepting electrons from FA.

## Discussion

The quantum yields (QYs) of solar to biomass for the photoautotrophic growth of *R. palustris* were first analyzed. It was calculated to be <1% for the photoexcited humus–driven photoautotrophic growth of *R. palustris* (Table 1). This value is lower compared to previously reported QY values for the growth or end-metabolite production of diverse autotrophic microorganisms driven by artificial photosensitizers such as Cadmium sulfide (CdS) nanoparticles [36]. This is most likely due to the photobleaching effect of humus upon long-term light exposure [33]. Furthermore, the presence of microplastics significantly increased the QY compared to their absence, indicating the role of microplastics as sacrificial quenchers. The biomass yield per mole of CO_2_ for the photoexcited humus–driven photoautotrophic growth of *R. palustris* was also calculated and found to be ~0.3–0.7. While this is lower than the previously reported biomass yield for the heterotrophic growth of *R. palustris*, it is similar to the biomass yield for photoautotrophic growth of *R. palustris* using thiosulfate as an electron donor (Table 1) [37]. It is important to note that the biomass yield for autotrophic growth may be underestimated due to the accumulation of a variety of carbon-containing pigments and storage compounds that cannot be accurately calculated as biomass [38].

**Table 1:** The quantum yield (QY)% and biomass yield for humus and microplastic-driven photoautotrophy of *R. palustris*.

	QY%^a^ *n* (electrons for CO_2_ reduction)/*n* (incident photons)	Biomass yield^b^ *n* (biomass)/n (CO_2_)
*R. palustris*-Na_2_S_2_O_3_		0.71 ± 0.07
*R. palustris*-FA	0.56 ± 0.18%	0.56 ± 0.31
*R. palustris*-FA + PLA	0.73 ± 0.04%	0.61 ± 0.10
*R. palustris*-FA + PET	0.72 ± 0.04%	0.35 ± 0.02
*R. palustris*-HA	0.18 ± 0.02%	0.68 ± 0.11
*R. palustris*-HA + PLA	0.60 ± 0.13%	0.54 ± 0.08
*R. palustris*-HA + PET	0.58 ± 0.02%	0.40 ± 0.11

a QY% was calculated by the number of the electrons for CO_2_ reduction divided by incident photons as described in M&M.

b Biomass yield was calculated by the increased biomass divided by consumed CO_2_ as described in M&M.

The analysis of the molecular weight of the microplastics revealed that PLA and PET depolymerized only when exposed to *R. palustris* in the presence of HA or FA. This suggests that the microplastics were likely oxidized by ROS generated by the photoexcited HA or FA, leading to their depolymerization. It was previously demonstrated that microplastics containing C–O or C=O, such as PLA and PET, are more susceptible to ROS attack than microplastics containing only C–C such as polystyrene or polyethylene [25]. This conclusion is further supported by the observed significant fluctuations in the abundances of C–O and C=O during the depolymerization of PLA or PET, as determined by FTIR and XPS analyses (Figs 4C and D andS3). Previous research has identified the depolymerization products of PET and PLA to contain ethylene glycol and lactate, respectively [23]. We also detected ethylene glycerol and lactate when PET and PLA were treated with FA or HA under illumination, respectively. Both ethylene glycol and lactate have been shown to be viable carbon sources for the photoheterotrophic growth of *R. palustris* [34, 35]. Therefore, it is reasonable to speculate that the enhanced photoautotrophic growth of *R. palustris* in the presence of microplastics can be attributed to the fact that the ROS-induced depolymerization products of microplastics serve as an additional carbon source for *R. palustris*, complementing CO_2_ and activating heterotrophic growth.

**Figure 5:**
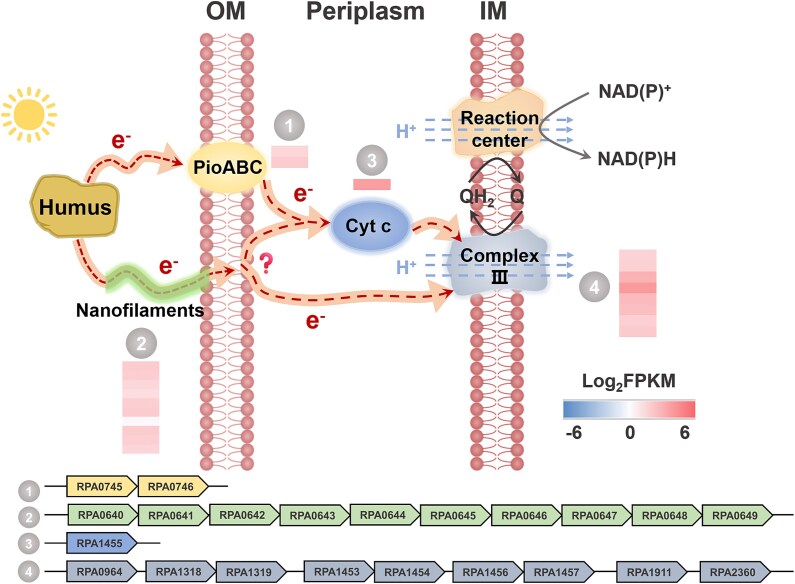
Proposed extracellular electron uptake pathways for the photoautotrophy of *R. palustris*. Genes involved in the pathways include those that encode (1) PioABC; (2) flagellar proteins; (3) a periplasmic cytochrome *c*; and (4) cytochrome *c* oxidases (Complex III). Inset heat maps show the average transcript abundance of corresponding genes from triplicate independent biohybrids presented as log_2_FPKM values (fragments per kilobase per million mapped reads).

In our transcriptomic analysis, we identified a potential extracellular electron uptake pathway in *R. palustris* using FA as the electron donor (Fig. 5). Previously, studies have reported that the outer membrane complex *pio*AB, which contains *c*-type cytochromes, plays a role in accepting electrons from extracellular Fe(II) minerals and cathodes in *R. palustris* [39, 40]. We observed upregulation of the gene cluster *pio*ABC in FA-driven photoautotrophic cells of *R. palustris* compared to that in thiosulfate-driven cells, suggesting that *pio*ABC may be involved in facilitating the uptake of photoexcited electrons produced from FA [41, 42]. Moreover, we found that 10 genes encoding conductive nanofilaments of flagella were upregulated in FA-driven photoautotrophic cells, indicating that flagella-mediated extracellular electron transfer could serve as an alternative pathway for the uptake of photoexcited electrons from FA [43–45]. Similar to electroautotrophy and electrosyntrophy by *R. palustris*, we observed upregulation of a gene encoding the periplasmic cytochrome *c*2 in FA-driven photoautotrophic cells, suggesting its role in mediating electron transfer from the periplasm to the inner membrane [46]. Furthermore, genes encoding components of the inner membrane cytochrome *c* oxidase (annotated as Complex III) were upregulated, indicating their involvement in transferring photoexcited electrons to the light-harvesting reaction center, which is responsible for cyclic photophosphorylation and energy conservation.

### Ecological implications

Anoxygenic phototrophs proliferate in sunlit anoxic environments, such as the water–soil interfaces, which were characterized as a biogeochemical hotspot [47]. Anoxygenic phototrophs exhibit remarkable metabolic versatility, utilizing diverse electron donors including dihydrogen, hydrogen sulfide, thiosulfate, elemental sulfur, and ferrous iron to support photoautotrophic growth and CO_2_ fixation. Humus stands out as a ubiquitous carbon pool in both terrestrial and aquatic ecosystems, and the widespread distribution and presence make it a particularly significant candidate as an electron donor for anoxygenic phototrophs. Humus concentrations in various aquatic and soil environments typically range from 0.1 to 30 mg/l, which aligns with the concentrations (5–30 mg/l) used in this study, underscoring the environmental relevance of our findings [48–50]. While humus has been implicated in mitigating CO_2_ emissions in aquatic and soil environments [5], its role as an electron donor upon photoexcitation for anoxygenic phototrophs in fixing CO_2_ has not been previously documented. Admittedly, establishing a definitive correlation between humus and CO_2_ emissions is challenging, given the complexity of microbial communities in natural environments and the limitations of batch culture experiments using only a model photosynthetic bacterium. Nonetheless, our results offer an ecophysiological explanation for how humus influences CO_2_ dynamics by serving as an electron donor for anoxygenic phototrophs.

In recent years, the intensification of plastic pollution has drawn attention to the role of microplastics in biogeochemical cycling [16]. Microplastics impact the biogeochemical carbon cycle and CO_2_ emissions through multifaceted mechanisms. For instance, they can alter soil particle aggregation and bulk density, thereby affecting the movement of nutrients, gases, and water, as well as microbial activity [17–19]. These changes influence whether carbon is sequestered in soil or metabolized and released into the atmosphere as CO_2_. Additionally, microplastics have been shown to modify the chlorophyll content of phytoplankton through poorly understood mechanisms, potentially affecting photosynthesis and microbial CO_2_ fixation [51]. Our study, based on batch culture experiments with a model photosynthetic bacterium, demonstrates how microplastics can influence CO_2_ flux when humus is photoexcited and serves as an electron donor for anoxygenic phototrophs. However, given that the microplastics selected for this study contain heteroatoms susceptible to ROS oxidation [25], further research is needed to determine whether microplastics with different chemical structures exhibit similar environmental behaviors.

## Supplementary Material

Supporting_Information_2_25_ycaf067

## Data Availability

The authors declare that all experimental data supporting the findings of this study are available within the article and its Supplementary Information files. The RNA-seq data generated in this study have been deposited in NCBI Trace Archive database under accession code PRJNA1160411.

## References

[1] Hayes MHB . Stevenson's humus. *Nature* 1983;303:835–6. 10.1038/303835b0

[2] Gramss G, Ziegenhagen D, Sorge S. Degradation of soil humic extract by wood- and soil-associated fungi, bacteria, and commercial enzymes. *Microb Ecol* 1999;37:140–51. 10.1007/s002489900138 9929402

[3] Grinhut T, Hadar Y, Chen Y. Degradation and transformation of humic substances by saprotrophic fungi: processes and mechanisms. *Fungal Biol Rev* 2007;21:179–89. 10.1016/j.fbr.2007.09.003

[4] Lipczynska-Kochany E . Humic substances, their microbial interactions and effects on biological transformations of organic pollutants in water and soil: a review. *Chemosphere* 2018;202:420–37. 10.1016/j.chemosphere.2018.03.104 29579677

[5] Valenzuela EI, Cervantes FJ. The role of humic substances in mitigating greenhouse gases emissions: current knowledge and research gaps. *Sci Total Environ* 2021;750:141677. 10.1016/j.scitotenv.2020.141677 33182214

[6] Lovley DR, Coates JD, Blunt-Harris EL. et al. Humic substances as electron acceptors for microbial respiration. *Nature* 1996;382:445–8. 10.1038/382445a0

[7] Lovley DR, Fraga JL, Coates JD. et al. Humics as an electron donor for anaerobic respiration. *Environ Microbiol* 1999;1:89–98. 10.1046/j.1462-2920.1999.00009.x 11207721

[8] Song Y, Huang R, Li L. et al. Humic acid-dependent respiratory growth of *Methanosarcina acetivorans* involves pyrroloquinoline quinone. *ISME J* 2023;17:2103–11. 10.1038/s41396-023-01520-y 37737251 PMC10579383

[9] Huang S, Chen M, Diao Y. et al. Dissolved organic matter acting as a microbial photosensitizer drives photoelectrotrophic denitrification. *Environ Sci Technol* 2022;56:4632–41. 10.1021/acs.est.1c07556 35319876

[10] Sharpless CM, Aeschbacher M, Page SE. et al. Photooxidation-induced changes in optical, electrochemical, and photochemical properties of humic substances. *Environ Sci Technol* 2014;48:2688–96. 10.1021/es403925g 24383955

[11] Ye J, Zhou S, Nealson KH. et al. Biophotoelectrochemistry: an emerging frontier for channeling photoelectric effect into darkness zone of soils and sediments. *Pedosphere* 2024;34:5–8. 10.1016/j.pedsph.2023.03.016

[12] Xiong J, Fischer WM, Inoue K. et al. Molecular evidence for the early evolution of photosynthesis. *Science* 2000;289:1724–30. 10.1126/science.289.5485.1724 10976061

[13] Johnston DT, Wolfe-Simon F, Pearson A. et al. Anoxygenic photosynthesis modulated proterozoic oxygen and sustained earth's middle age. *Proc Natl Acad Sci* 2009;106:16925–9. 10.1073/pnas.0909248106 19805080 PMC2753640

[14] Ozaki K, Thompson KJ, Simister RL. et al. Anoxygenic photosynthesis and the delayed oxygenation of earth’s atmosphere. *Nature*. *Communications* 2019;10:10. 10.1038/s41467-019-10872-z PMC6616575 31289261

[15] Hohmann-Marriott MF, Blankenship RE. Evolution of photosynthesis. *Annu Rev Plant Biol* 2011;62:515–48. 10.1146/annurev-arplant-042110-103811 21438681

[16] Huang W, Xia X. Element cycling with micro(nano)plastics. *Science* 2024;385:933–5. 10.1126/science.adk9505 39208108

[17] Stubbins A, Law KL, Muñoz SE. et al. Plastics in the earth system. *Science* 2021;373:51–5. 10.1126/science.abb0354 34210876

[18] Zhou J, Gui H, Banfield CC. et al. The microplastisphere: biodegradable microplastics addition alters soil microbial community structure and function. *Soil Biol Biochem* 2021;156:108211. 10.1016/j.soilbio.2021.108211

[19] Rillig MC, Kim SW, Zhu Y-G. The soil plastisphere. *Nat Rev Microbiol* 2023;22:64–74. 10.1038/s41579-023-00967-2 37697003 PMC7615554

[20] Romera-Castillo C, Pinto M, Langer TM. et al. Dissolved organic carbon leaching from plastics stimulates microbial activity in the ocean. *Nat Commun* 2018;9:9. 10.1038/s41467-018-03798-5 29651045 PMC5897397

[21] Sheridan PO, Meng Y, Williams TA. et al. Recovery of lutacidiplasmatales archaeal order genomes suggests convergent evolution in thermoplasmatota. *Nature*. *Communications* 2022;13:13. 10.1038/s41467-022-31847-7 PMC9287336 35840579

[22] Tang X, Han X, Sulaiman NHM. et al. Recent advances in the photoreforming of plastic waste: principles, challenges, and perspectives. *Ind Eng Chem Res* 2023;62:9032–45. 10.1021/acs.iecr.3c00809

[23] Uekert T, Kasap H, Reisner E. Photoreforming of nonrecyclable plastic waste over a carbon nitride/nickel phosphide catalyst. *J Am Chem Soc* 2019;141:15201–10. 10.1021/jacs.9b06872 31462034 PMC7007225

[24] Zan L, Fa W, Wang S. Novel photodegradable low-density polyethylene−TiO_2_ nanocomposite film. *Environ Sci Technol* 2006;40:1681–5. 10.1021/es051173x 16568787

[25] Zhou G, Xu H, Song H. et al. Photocatalysis toward microplastics conversion: a critical review. *ACS Catal* 2024;14:8694–719. 10.1021/acscatal.4c01449

[26] Sun D-W, Chen K-L, Huang J-H. Benzenesulfonyl chloride-incorporated g-C_3_N_4_ for photocatalytic hydrogen generation by using the hydrolysate of poly(lactic acid) as sacrificial reagent. *Appl Catal A Gen* 2021;628:118397. 10.1016/j.apcata.2021.118397

[27] Larimer FW, Chain P, Hauser L. et al. Complete genome sequence of the metabolically versatile photosynthetic bacterium *Rhodopseudomonas palustris*. *Nat Biotechnol* 2003;22:55–61. 10.1038/nbt923 14704707

[28] Huang JJ, Heiniger EK, McKinlay JB. et al. Production of hydrogen gas from light and the inorganic electron donor thiosulfate by *Rhodopseudomonas palustris*. *Appl Environ Microbiol* 2010;76:7717–22. 10.1128/aem.01143-10 20889777 PMC2988585

[29] Gomes RS, Fernandes AN, Waldman WR. How to measure polymer degradation? An analysis of authors' choices when calculating the carbonyl index. *Environ Sci Technol* 2024;58:7609–16. 10.1021/acs.est.3c10855 38624261

[30] Chen M, Cai Q, Chen X. et al. Anthraquinone-2-sulfonate as a microbial photosensitizer and capacitor drives solar-to-N_2_O production with a quantum efficiency of almost unity. *Environ Sci Technol* 2022;56:5161–9. 10.1021/acs.est.1c08710 35312317

[31] Bai W, Ranaivoarisoa TO, Singh R. et al. N-butanol production by *Rhodopseudomonas palustris* tie-1. *Commun Biol* 2021;4:4. 10.1038/s42003-021-02781-z 34732832 PMC8566592

[32] Guan X, Erşan S, Hu X. et al. Maximizing light-driven CO_2_ and N_2_ fixation efficiency in quantum dot–bacteria hybrids. *Nat Catal* 2022;5:1019–29. 10.1038/s41929-022-00867-3 36844635 PMC9956923

[33] Niu X-Z, Liu C, Gutierrez L. et al. Photobleaching-induced changes in photosensitizing properties of dissolved organic matter. *Water Res* 2014;66:140–8. 10.1016/j.watres.2014.08.017 25201337

[34] Brown B, Wilkins M, Saha R. *Rhodopseudomonas palustris*: a biotechnology chassis. *Biotechnol Adv* 2022;60:108001. 10.1016/j.biotechadv.2022.108001 35680002

[35] Li M, Ning P, Sun Y. et al. Characteristics and application of *Rhodopseudomonas palustris* as a microbial cell factory. *Front Bioeng Biotechnol* 2022;10:10. 10.3389/fbioe.2022.897003 PMC9133744 35646843

[36] Cestellos-Blanco S, Zhang H, Kim JM. et al. Photosynthetic semiconductor biohybrids for solar-driven biocatalysis. *Nat Catal* 2020;3:245–55. 10.1038/s41929-020-0428-y

[37] McKinlay JB, Harwood CS. Carbon dioxide fixation as a central redox cofactor recycling mechanism in bacteria. *Proc Natl Acad Sci* 2010;107:11669–75. 10.1073/pnas.1006175107 20558750 PMC2900684

[38] Holland AD, Dragavon JM, Sigee DC. Intrinsic autotrophic biomass yield and productivity in algae: experimental methods for strain selection. *Biotechnol J* 2011;6:572–83. 10.1002/biot.201000260 21381200

[39] Guzman MS, Rengasamy K, Binkley MM. et al. Phototrophic extracellular electron uptake is linked to carbon dioxide fixation in the bacterium *Rhodopseudomonas palustris*. *Nat Commun* 2019;10:1355. 10.1038/s41467-019-09377-6 30902976 PMC6430793

[40] Bose A, Gardel EJ, Vidoudez C. et al. Electron uptake by iron-oxidizing phototrophic bacteria. *Nat Commun* 2014;5:3391. 10.1038/ncomms4391 24569675

[41] Huang L, Liu X, Zhang Z. et al. Light-driven carbon dioxide reduction to methane by *Methanosarcina barkeri* in an electric syntrophic coculture. *ISME J* 2022;16:370–7. 10.1038/s41396-021-01078-7 34341507 PMC8776907

[42] Liu X, Huang L, Rensing C. et al. Syntrophic interspecies electron transfer drives carbon fixation and growth by *Rhodopseudomonas palustris* under dark, anoxic conditions. *Science*. *Advances* 2021;7:eabh1852. 10.1126/sciadv.abh1852 PMC11057707 34215588

[43] Lovley DR . Syntrophy goes electric: direct interspecies electron transfer. *Ann Rev Microbiol* 2017;71:643–64. 10.1146/annurev-micro-030117-020420 28697668

[44] Rotaru A-E, Shrestha PM, Liu F. et al. A new model for electron flow during anaerobic digestion: direct interspecies electron transfer to methanosaeta for the reduction of carbon dioxide to methane. *Energy Environ Sci* 2014;7:408–15. 10.1039/c3ee42189a

[45] Venkidusamy K, Megharaj M, Schröder U. et al. Electron transport through electrically conductive nanofilaments in *Rhodopseudomonas palustris* strain rp2. *RSC Adv* 2015;5:100790–8. 10.1039/c5ra08742b

[46] Ren G, Ye J, Hu Q. et al. Growth of electroautotrophic microorganisms using hydrovoltaic energy through natural water evaporation. *Nat Commun* 2024;15:15. 10.1038/s41467-024-49429-0 38862519 PMC11166942

[47] Cai Y-J, Liu Z-A, Zhang S. et al. Microbial community structure is stratified at the millimeter-scale across the soil–water interface. *ISME Commun* 2022;2:53. 10.1038/s43705-022-00138-z 37938662 PMC9723559

[48] Wei Y, Li H, Liu G. et al. Insights into the spectral characteristics and sources of dissolved organic matter in a water supply reservoir. *Bull Environ Contam Toxicol* 2025;114:1–10. 10.1007/s00128-024-03994-8 39831970

[49] Qi Y, Xie Q, Wang J-J. et al. Deciphering dissolved organic matter by fourier transform ion cyclotron resonance mass spectrometry (ft-icr ms): from bulk to fractions and individuals. *Carbon*. *Research* 2022;1:1–22. 10.1007/s44246-022-00002-8

[50] Philippe A, Schaumann GE. Interactions of dissolved organic matter with natural and engineered inorganic colloids: a review. *Environ Sci Technol* 2014;48:8946–62. 10.1021/es502342r 25082801

[51] Amaneesh C, Anna Balan S, Silpa PS. et al. Gross negligence: impacts of microplastics and plastic leachates on phytoplankton community and ecosystem dynamics. *Environ Sci Technol* 2022;57:5–24. 10.1021/acs.est.2c05817 36534053

