# Supplementary material for: Anoxygenic photoautotrophy driven by humus and microplastics in a photosynthetic bacterium
Source: ISME Commun. 2025 Apr 18;5(1):ycaf067. doi: 10.1093/ismeco/ycaf067 (PMC12066414; doi:10.1093/ismeco/ycaf067)
Supplement: Supporting_Information_2_25_ycaf067 [file supporting_information_2_25_ycaf067.pdf]

Supporting Information for

Anoxygenic photoautotrophy driven by humus and microplastics in a photosynthetic bacterium

Yutong Li, Kongyuang Qu, Jianming Yang, Shuguang Wang*, Zhen Yan*

This PDF file includes:

Figures S1 to S6

Tables S1 to S3

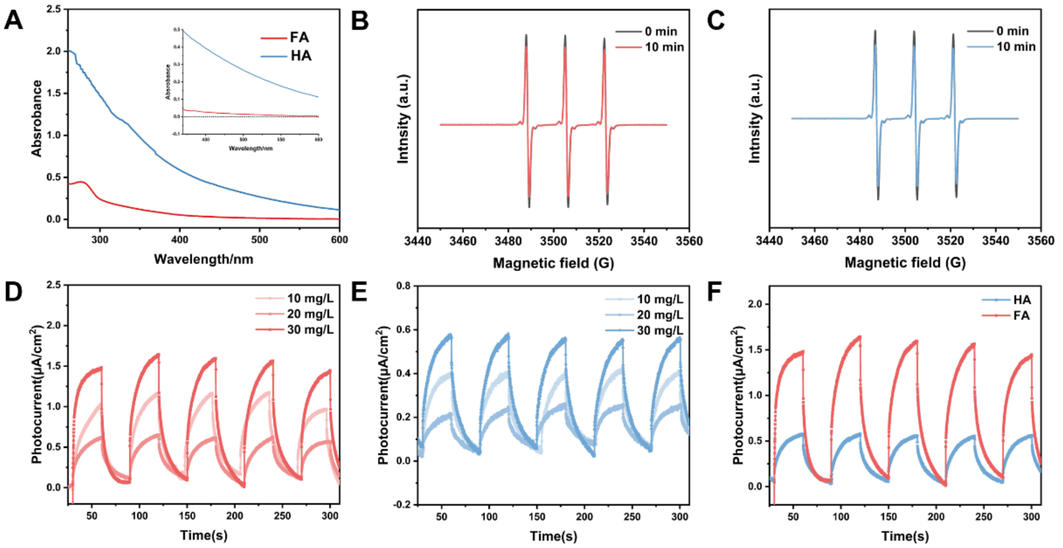

Figure S1. Characterization of the photochemical properties of FA and HA. (a)UV-vis spectra of FA and HA aqueous solution (30 mg/L) (Inset is the UV-vis spectrum of FA and HA aqueous solution at wavelengths of 400-600 nm); EPR spectra for h^+ of (b) FA and (c) HA upon illumination of 10 min; *I*-*t* curves of (d) FA and (e) HA at different concentration with a light/off cycle (30/30 s), and (f) the comparison between FA and HA at 30 mg/L.

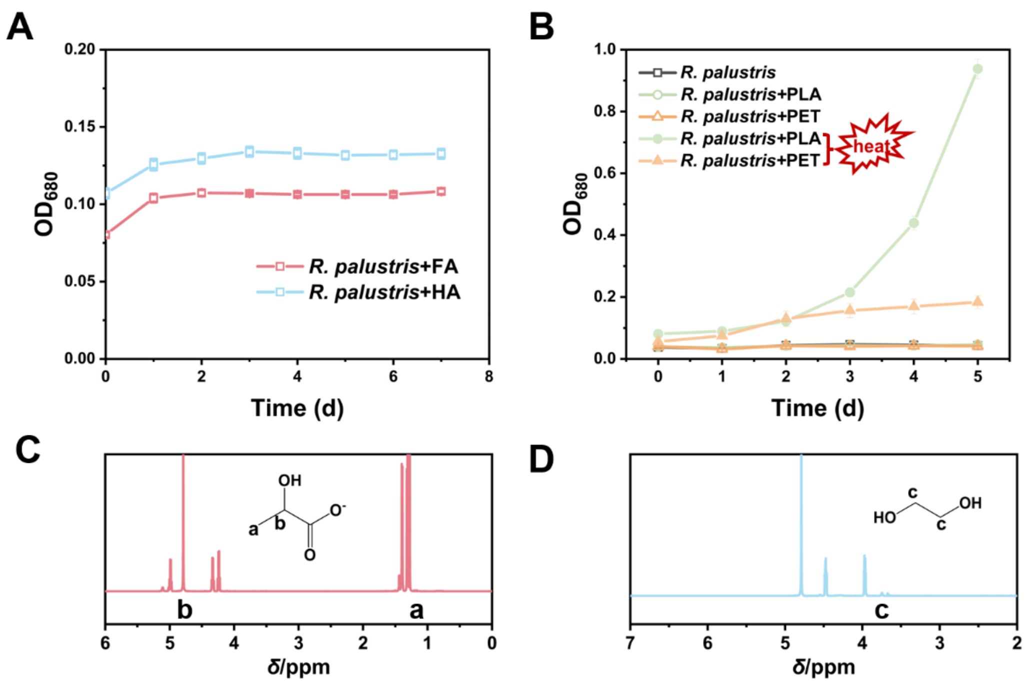

Figure S2. Analysis of humus and microplastics as a carbon source for the photoautotrophic growth of *R. palustris*. (a) Growth curves for the culture of *R. palustris* with 30 mg/L FA or HA in the absence of CO_2 in the headspace. (b) Growth curves for the culture of *R. palustris* amended with 100 mg/L PLA or PET with heat pretreatment or not in the absence of FA or HA in the medium; ^{1}H NMR spectra of the pyrolysis products of (c) PLA and (d) PET.

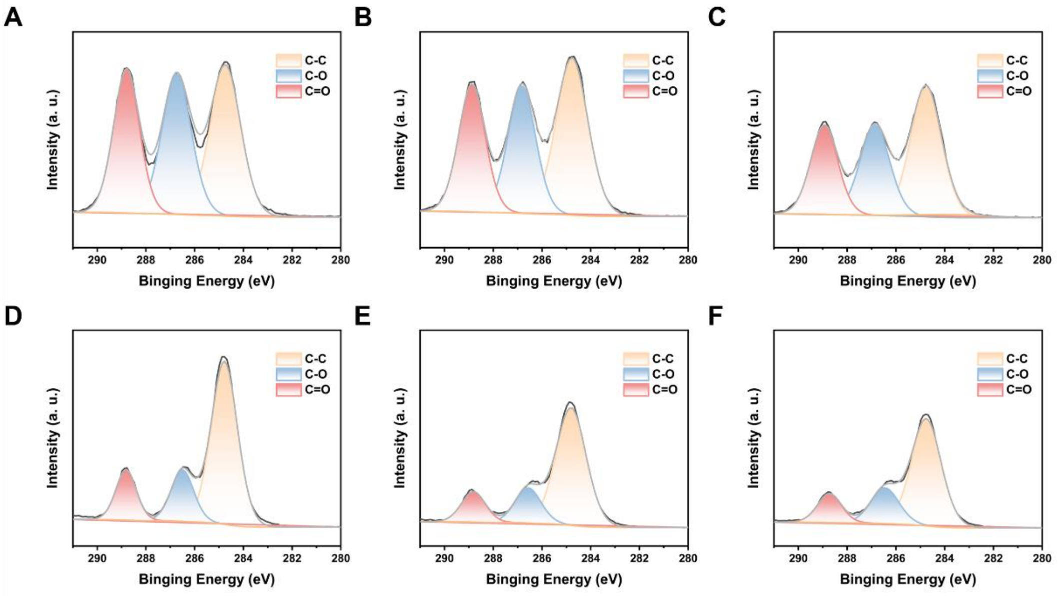

Figure S3. XPS spectra of C 1s. Untreated (a) PLA and (d) PET; (b) PLA and (e) PET with *R. palustris*+HA treatment; (c) PLA and (f) PET with *R. palustris*+FA treatment.

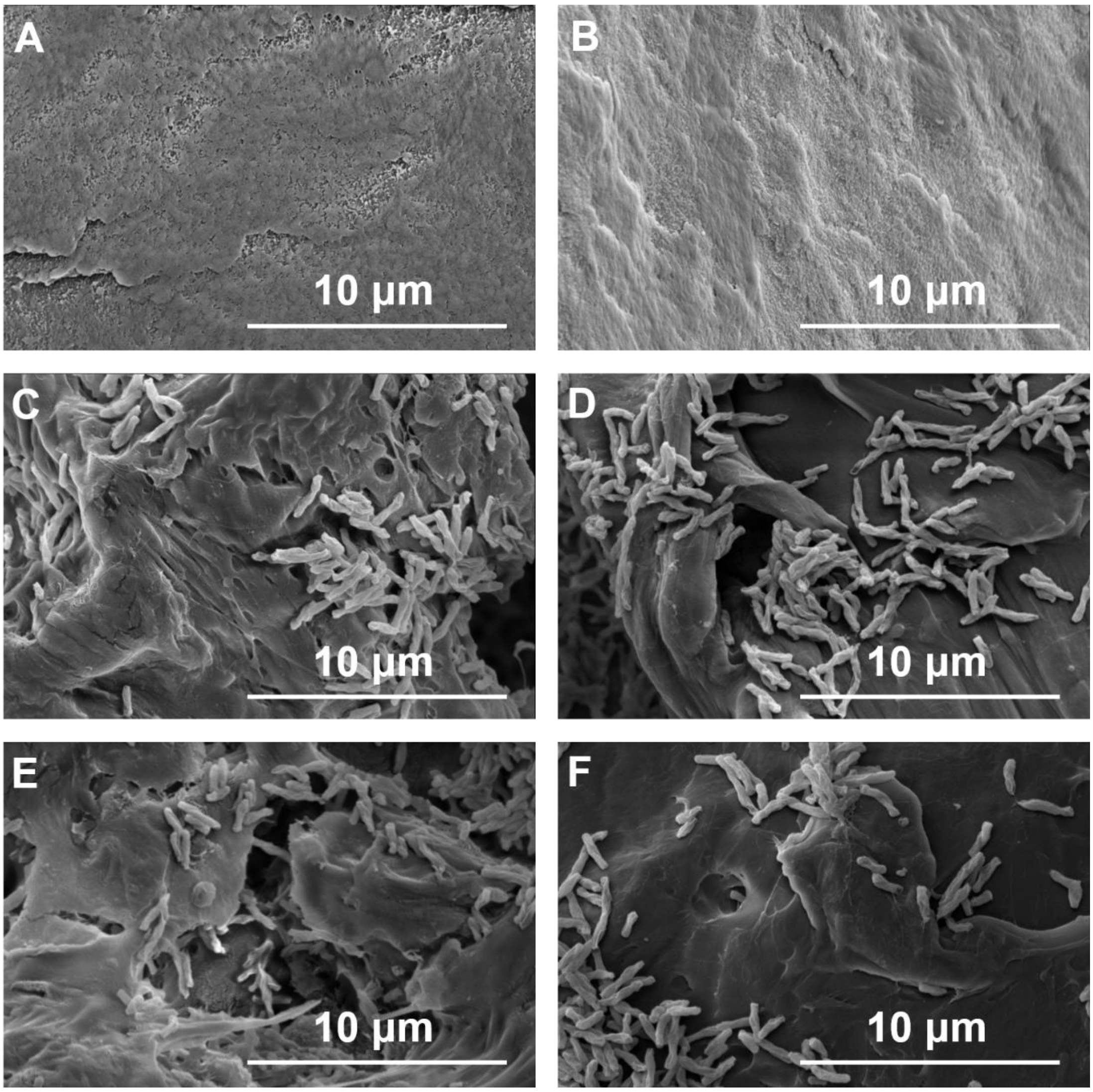

Figure S4. SEM images of PLA and PET with different incubation. (a) PLA and (b) PET; (c) PLA and (d) PET incubated with *R. palustris*+HA for 6 days; (e) PLA and (f) PET incubated with *R. palustris*+FA for 6 days.

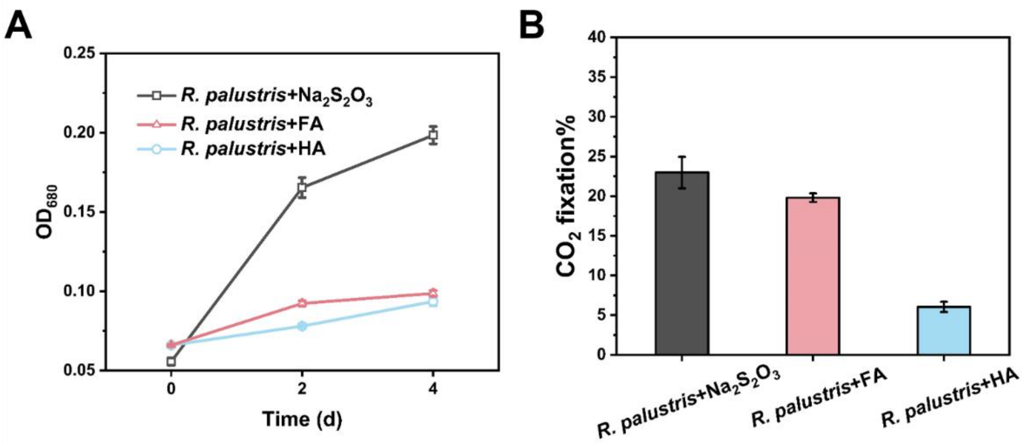

Figure S5. Comparison of the photoautotrophic growth of *R. palustris* with sodium thiosulfate (4 mM) or humus (30 mg/L FA or HA) as an electron donor. (a) Growth curves; (b) CO_2 fixation rate.

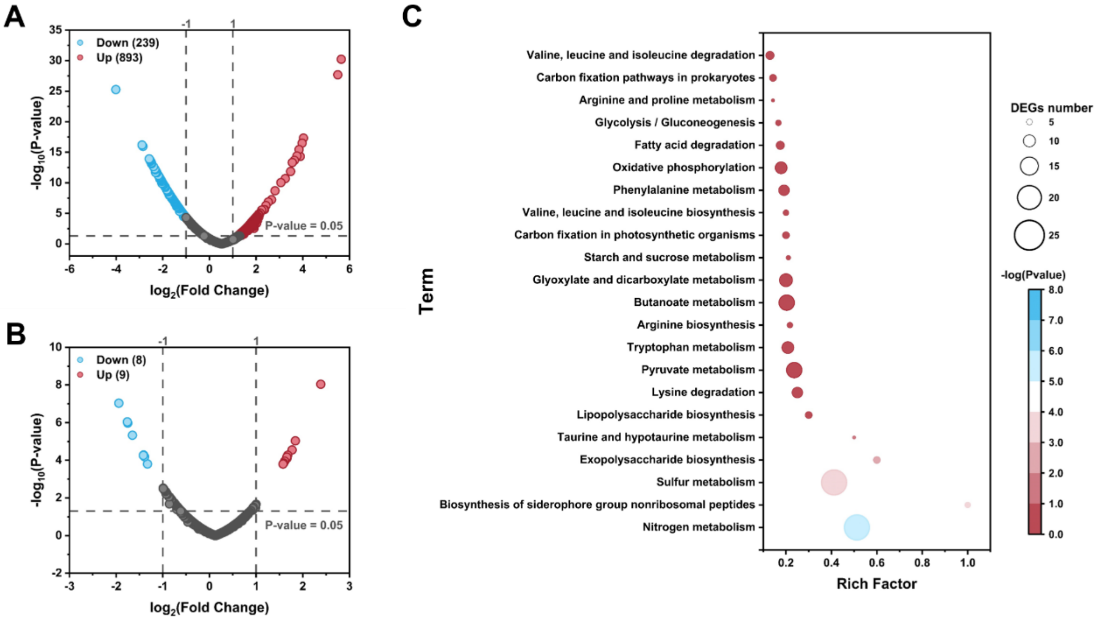

Figure S6. Transcriptomic analyses of *R. palustris*. Differential gene volcano graph (a) of the *R. palustris*+FA compared with *R. palustris*, and (b) of the *R. palustris*+FA+PLA compared with *R. palustris*+FA (Dashed lines represent the gating of fold change (FC)>2.0 and p<0.05); (c) The metabolic pathway enrichment map of *R. palustris*+FA compared with *R. palustris*. p<0.05 was used as a filter condition. KEGG terms in the classification were screened according to p<0.05. Bubble color and size indicate -log(p-*value*) and the number of genes in KEGG pathways, respectively.

Table S1. EIS fitting results of *R. palustris* and biohybrid under light or dark condition.

	R_1/ ($\Omega\cdot cm^2$)	$Q_1\times10^{-4}$/ ($\Omega^{-1}s^n\cdot cm^{-2}$)	R_2/ ($\Omega\cdot cm^2$)	$Q_2\times10^{-3}$/ ($\Omega^{-1}s^n\cdot cm^{-2}$)	R_3/ ($\Omega\cdot cm^2$)
R. palustris-D	17.78	3.69	382.6	5.43	318.8
R. palustris -L	17.78	3.83	389.9	5.13	342.1
R. palustris +HA-D	13.65	3.17	299.8	6.53	307.8
R. palustris +HA-L	13.92	2.92	271.5	6.00	316.8
R. palustris +FA-D	15.09	3.58	268.8	6.63	368.9
R. palustris +FA-L	15.12	3.53	254.7	6.70	399.5

Table S2. Chemical composition of PLA and PET with different treatment.

	C=O%	O/C%	Molecular weight		
			M_P	M_N	M_W
PLA	19.02	0.63	56933	22065	73645
PLA-*R. palustris*	-	-	52411	20463	71793
PLA-*R. palustris*+HA	17.65	0.60	34287	14074	43455
PLA- *R. palustris*+FA	16.93	0.56	33693	12235	43004
PET	11.56	0.37	57139	37308	72016
PET- *R. palustris*	-	-	57139	36843	71274
PET- *R. palustris*+HA	10.10	0.36	45602	30137	60023
PET- *R. palustris*+FA	10.42	0.36	35945	24502	57157

Table S3. Significantly upregulated genes involved in extracellular electron transfer and CO_2 fixation.

	Gene_ID	Log_2(FC)	*Pvalue*	Annotation
**cytochrome *c***	TX73_007475	3.840644675	3.64E-16	cytochrome *c*
**complex III**	TX73_011925	1.726293183	0.001268957	cytochrome *b*
	TX73_009845	1.787696075	0.000933028	cytochrome *b*/*b6* domain-containing protein
	TX73_007485	3.057574541	9.34E-11	CbbQ/NirQ/NorQ/GpvN family protein
	TX73_007480	4.029388331	4.66E-18	cbb3-type cytochrome *c* oxidase subunit I
	TX73_007470	2.663555385	5.87E-08	cytochrome *c* oxidase subunit IV family protein
	TX73_007465	2.522780431	1.42E-07	cytochrome *c* oxidase subunit 3 family protein
	TX73_006765	1.801325975	0.00041964	cytochrome *d* ubiquinol oxidase subunit II
	TX73_006760	2.065316757	3.54E-05	cytochrome ubiquinol oxidase subunit I
**NADH-quinone oxidoreductase**	TX73_003790	1.609111701	0.003347621	NADH-quinone oxidoreductase subunit NuoF
**pioABC**	TX73_003855	1.533096815	0.004381554	DmsE family decaheme *c*-type cytochrome

	TX73_003850	2.030774773	3.46E-05	MtrB/PioB family decaheme-associated outer membrane protein
flagella	TX73_003365	2.014664917	6.75E-05	flagellin
	TX73_003360	1.883689196	0.00015587	flagellar hook-associated protein FlgK
	TX73_003355	1.52635828	0.007883428	flagellar protein FlgN
	TX73_003350	1.289344658	0.04167338	hypothetical protein
	TX73_003345	1.854066374	0.000300695	flagellar hook protein FlgE
	TX73_003340	1.969605785	9.44E-05	FlgD immunoglobulin-like domain containing protein
	TX73_003335	0.223408377	0.384756074	hypothetical protein
	TX73_003330	1.990248409	0.000191967	flagellar protein FliS
	TX73_003325	1.862028174	0.000200147	flagellar filament capping protein FliD
	TX73_003320	1.570103041	0.003594619	flagellin
CO_2 fixation	TX73_024105	-1.361812817	7.17E-07	fructose-bisphosphate aldolase class II
	TX73_008020	-2.152848482	1.17E-11	CbbX protein
	TX73_008010	-2.319496203	7.50E-13	form I ribulose bisphosphate carboxylase large subunit

	TX73_008015	-2.582944333	1.22E-14	ribulose bisphosphate carboxylase small subunit
Light-harvest	TX73_013740	-1.605592757	3.02E-08	light-harvesting protein
	TX73_007850	-1.502578521	1.21E-07	light-harvesting antenna LH1, alpha subunit
	TX73_007670	-1.510723445	1.09E-07	light-harvesting protein
	TX73_013745	-1.452365938	2.28E-07	light-harvesting antenna LH1, beta subunit
	TX73_007845	-1.434623646	2.85E-07	light-harvesting antenna LH1, beta subunit
	TX73_015600	-1.381165181	6.09E-07	light-harvesting protein
	TX73_015585	-1.194267373	6.06E-06	light-harvesting antenna LH1, beta subunit
	TX73_007965	-1.01935728	3.99E-05	photosynthetic complex assembly protein PuhC
Sox	TX73_023190	-1.912365981	3.95E-10	thiosulfate oxidation carrier complex protein SoxZ
	TX73_023200	-1.518316887	1.01E-07	sulfur oxidation c-type cytochrome SoxA
	TX73_023195	-1.332984713	1.05E-06	thiosulfate oxidation carrier protein SoxY
	TX73_023205	-1.080706499	2.16E-05	sulfur oxidation c-type cytochrome SoxX
